# A framework of the desirable features of guideline implementation tools (GItools): Delphi survey and assessment of GItools

**DOI:** 10.1186/s13012-014-0098-8

**Published:** 2014-08-05

**Authors:** Anna R Gagliardi, Melissa C Brouwers, Onil K Bhattacharyya

**Affiliations:** University Health Network, Toronto, ON Canada; McMaster University, Hamilton, ON Canada; Women’s College Hospital, Toronto, ON Canada

**Keywords:** Guidelines, Implementation

## Abstract

**Background:**

Guidelines are the foundation for healthcare planning, delivery and quality improvement but are not consistently implemented. Few guidelines are accompanied by guideline implementation tools (GItools). Users have requested GItools, and developers have requested guidance on how to develop GItools. First it is necessary to characterize GItools. The purpose of this research was to generate a framework of desirable features of GItools.

**Methods:**

Items representing desirable GItool features were generated by a cross-sectional survey of the international guideline community. Items were confirmed by 31 guideline developers, implementers and researchers in a two-round Delphi survey administered on the Internet. The resulting GItool framework was applied with a sample of GItools accompanying guidelines identified in the National Guideline Clearinghouse.

**Results:**

The cross-sectional survey was completed by 96 respondents from Australia, Canada, the United Kingdom, the United States, The Netherlands, and various other countries. Seven of nine items were rated by the majority as desirable. A total of 31 panelists from 10 countries including Australia, Canada, Germany, New Zealand, Peru, Saudi Arabia, Spain, the United Kingdom, and the United States took part in a two-round Delphi survey. Ten items achieved consensus as desirable GItool features in round #1, and two additional items in round #2. A total of 13 GItools for Resource Planning, Implementation and Evaluation were identified among 149 guidelines on a variety of clinical topics (8.7%). Many GItools did not possess features considered desirable.

**Conclusions:**

Inclusion of higher quality GItools in guidelines is needed to support user adoption of guidelines. The GItool framework can serve as the basis for evaluating and adapting existing GItools, or developing new GItools. Further research is needed to validate the framework, develop and implement instruments by which developers can apply the framework, and specify which guidelines should be accompanied by GItools.

## Background

Guidelines began to be widely produced in the 1980s by government agencies and professional societies to address concerns about practice variations and the safety of biomedical technology [1]. They continue to proliferate due to increased pressure by the public and payers to provide optimal care [2]. Guidelines are seen as the ‘foundation of efforts to improve healthcare’ at the micro (individual clinician or patient), meso (group, institution) and macro (economic, political) level by informing policy, planning, delivery and evaluation [2],[3]. Directories such as the National Guideline Clearinghouse (www.guideline.gov) were created to enhance access to guidelines, and guideline developers, implementers and researchers formed the Guidelines International Network (www.g-i-n.net) to advance guideline development and implementation through knowledge sharing, research and collaborative efforts. The recently issued Checklist for Guideline Development provides an 18-category framework by which to plan and undertake guideline development and implementation [4]. The Checklist is based on considerable research that demonstrates how much the guideline enterprise has evolved. It draws upon AGREE II, GRADE and GLIA, which offer principles, standards and criteria for developing robust and trustworthy guidelines in which the quantity and quality of evidence is apparent, and recommendations are clearly worded and actionable [5]-[7]. Numerous appraisal tools have also been developed to help guideline users assess the quality of guidelines [8].

Despite these improvements in the process of guideline development and in the quality of guidelines, their use remains inconsistent, leading to sub-optimal system, organization and clinical/patient outcomes. An analysis of population-based data reflecting 439 recommendations for 30 conditions spanning preventive, acute and chronic services found that 54.9% of patients in the United States received guideline-recommended care [9]. A similar study in the United Kingdom found no improvement in patient care following release of guidelines for a variety of conditions [10]. Surveys of guideline developers worldwide found that many do not implement their guidelines and believe that this is the responsibility of guideline users [11],[12]. We interviewed international guideline developers about their implementation practices and challenges [13]. This revealed considerable variability in funding, staffing models and implementation planning approaches. Most developers disseminated guidelines on web sites and in academic journals but did not have funding for more elaborate, proactive or targeted implementation activities.

More recent studies provide some insight on factors that challenge guideline implementation among users. A systematic review found wide variation across different guidelines in reported awareness (28% to 100%), agreement (12.7% to 97%), adoption (11% to 97.4%) and adherence (16.7% to 84.1%) and, even when awareness or agreement were high, reported adoption and adherence were comparatively lower [14]. Focus groups and interviews with clinicians also found that even with the best of intentions to implement a guideline, health professionals were frustrated and at a loss as to how to achieve that in practice [15]. These studies reveal that guideline quality, or awareness of and agreement with guidelines may not pose major hurdles to guideline uptake. Instead, guideline users require support for implementation. Others have also suggested that guideline developers pay greater attention to providing clinicians with tools that support implementation such as assessment instruments to identify guideline-specific barriers and corresponding implementation strategies, or checklists that could be integrated into clinical decision support systems [16]. This idea is supported by cognitive science theory, which suggests that guidelines may be difficult to use because they present complex information recommending action that may not match patient, provider and organizational contextual circumstances that often interact to challenge guideline implementation and use [17]. Empirical research supports this theory. A systematic review found that compliance with 143 guidelines was greater for those that included instructions or resources for implementing the recommendations, a tactic to reduce the complexity of information, and two trials found that guidelines containing instructions or resources were implemented more than usual guidelines [18]-[20].

There are several types of guideline implementation tools (GItools) that provide users with instructions or resources for guideline implementation [21]. These include support for resource planning (*e.g*., human resource planning, costing models); implementation (clinical algorithms, chart documentation forms or checklists, mobile device resources, pocket guides or reference cards, slide presentations, staff training material); and evaluation (indicators/performance measures, benchmarks, audit instructions). Our interviews with 30 guideline developers or implementers from government and professional societies in seven countries found that few had developed GItools [13]. However, they described a demand for GItools among target users of their guidelines and requested guidance for developing GItools. We examined guideline development instructional manuals for information on how to develop GItools but most were lacking in this regard, highlighting the need to develop resources that support GItool adaptation or development [22]. This was recently confirmed by a more expansive review of guideline development instructional resources [4].

GItools can support guideline implementation, but our research found that they are not consistently offered in or with guidelines [13],[21]. Developers have requested guidance on how to develop GItools. Before developing such guidance, it is first necessary to better characterize the ideal characteristics of GItools. The primary purpose of this research was to generate a framework of the desirable features of GItools that could serve as the basis by which to evaluate and adapt existing GItools, or develop new GItools. A secondary or related purpose was to apply the framework to describe GItools included in, or accompanying, a selection of guidelines as a first measure of the status of the field.

## Methods

### Overall approach

Items reflecting the desirable features of GItools were first generated and vetted with a cross-sectional survey of international guideline developers, implementers and researchers based on standard descriptive survey methods [23]. Then items were confirmed and expanded by a panel of international guideline developers, implementers and researchers through a two-stage Delphi process [24]. The resulting framework was used to describe the features of GItools accompanying a selection of guidelines. For this research, GItools were defined as ‘information within or accompanying guidelines that helps users implement the recommendations.’ The definition was purposefully broad to be as inclusive as possible. Three types of GItools were considered and defined as follows: Resource planning (equipment or technology needed; industrial standards; policies governing their use; type and number of health professionals needed to deliver services; staff education, training or competencies; anticipated changes in workflow during or after adoption; associated costs); Implementation (assessing of individual, organizational and system barriers associated with adoption; selecting and tailoring implementation strategies that address barriers; point-of-care tools in which recommendations are embedded); and Evaluation (performance measures/quality indicators; instructions on how to develop indicators; relevant benchmarks; instructions by which individuals or organizations can assess baseline and/or post-adoption performance). Ethics approval for this research was established at the University Health Network, Toronto, Canada.

### Cross-sectional survey to generate and rate candidate GItool features

A survey was drafted to generate and rate the desirability of candidate GItool features. Candidate GItool features were initially informed by items considered fundamental to the transparency, reliability and validity of evaluation or measurement instruments [25]. These included statements about objectives and target users, and details about development, underlying evidence, and testing. Section One defined GItools as noted above, and provided URL links to two examples for each of the three types of GItools (Resource planning, Implementation, Evaluation) so that respondents could familiarize themselves with various GItools (Table 1). Section Two listed nine GItool features. Respondents were asked to rate the desirability of each feature for assessing or developing GItools on a 7-point Likert-type scale ranging from ‘strongly disagree’ to ‘strongly agree.’ Free text options were included for comments on the wording or content of items, and to suggest additional items. The survey was reviewed by the research team, who suggested minor edits to wording.

**Table 1:** **GItools provided as examples in cross-sectional survey**

Type of GItool	Examples
Resource Implications	Guide to the Implementation of Stroke Unit Care, http://strokebestpractices.ca/wp-content/uploads/2010/11/CSS-Stroke-Unit-Resource_EN-Final2-for-print.pdf
	Pan-Canadian Health Human Resource Planning Toolkit, http://www.nshrf.ca/initiatives/initiatives/pan-canadian-health-human-resources-planning-toolkit
Implementation	Canadian Respiratory Guidelines Toolkit, http://www.respiratoryguidelines.ca/toolkit
	Canadian Guideline for Safe and Effective Use of Opioids for Chronic Non-cancer Pain Opioid Manager – Opioid Manager (for EMR), http://nationalpaincentre.mcmaster.ca/opioidmanager/
Evaluation	Canadian Stroke Strategy Performance Measurement Manual, http://www.strokebestpractices.ca/wp-content/uploads/2012/07/CSS-Performance-Manual-2008_EN.pdf
	Canadian Association of Radiologists: Maximizing the effectiveness of clinical audits, http://www.car.ca/uploads/education lifelong learning/201101_en_car_guide_clinicalaudit.pdf

The survey was administered using an Internet application to members of the Guidelines International Network (G-I-N). G-I-N at the time included 107 individual and 86 organizations members from 45 countries. Members include guideline developers, implementers and researchers. The G-I-N secretariat announced the survey to G-I-N members via email in July 2012. A reminder was sent at two weeks and four weeks according to the tailored design method [26]. Response rate was not calculated since a denominator could not be accurately established. Response frequencies were calculated, items rated ‘agree’ or ‘strongly agree’ by 65% or more of respondents were noted, and comments were summarized.

### Delphi consensus process to confirm desirable GItool features

A survey was developed to establish consensus on desirable GItool features. GItools were defined as noted above along with a URL link to a web site featuring examples of GItools (http://giranet.org). All nine GItool features considered in the previous cross-sectional survey were included. Additional items were generated at a meeting of Canadian guideline developers in Toronto, Canada, on May 10, 2013. The meeting was held to solicit input on how to characterize GItools. All developers of Canadian guidelines were initially identified by searching the Agency for Healthcare Research and Quality’s Guideline Clearinghouse (http://www.guideline.gov) and searching MEDLINE for [Canada AND guidelines as topic]. Participants were recruited to attend the meeting by email invitation. A total of 28 participants were divided into 5 small groups that each reviewed a different GItool with the nine GItool features. Each small group reported the findings of their review. Then the full assembly discussed the desirable features of GItools. As a result, 7 additional GItool features were recommended and added to the original 9 for a total of 16 items in the survey. Each was to be rated on a 7-point Likert-type scale ranging from ‘strongly disagree’ to ‘strongly agree.’ A free text option was included for additional items suggested by participants. A panel was assembled comprised of 31 guideline developers, implementers or researchers from countries with well-established guideline programs. They were selected from among G-I-N members for their expertise, experience or expressed interest in GItools. They were contacted by email to explain the process and confirm their participation. The survey was administered using an Internet application. A reminder was sent at two weeks and four weeks [26]. Frequencies of panel responses were calculated to establish the degree of consensus for each item. Standard Delphi protocol suggests that two or three rounds of rating with agreement by two thirds of panelists will prevent respondent fatigue and drop-out while establishing reasonable consensus [27],[28]. The following consensus categories were applied: strong consensus for acceptance (20 or more panel members agreed or strongly agreed by choosing 6 or 7), strong consensus for exclusion (20 or more panel members disagreed or strongly disagreed by choosing 1 or 2) and unclear consensus (20 or more panel members chose 3, 4, 5 or ‘Not Sure’). Newly suggested criteria were noted. A Round #1 report was prepared summarizing anonymized rating frequencies and comments, grouping criteria by consensus category, and listing newly suggested criteria. The Round #1 report was returned to panelists by email along with a link to the Round #2 questionnaire formatted similar to that in Round #1 for rating of items that had not yet achieved consensus for inclusion or exclusion. Similar analysis was performed to summarize Round #2 results. The Delphi process took place during June and July of 2013. This generated a list of GItool features considered desirable according to the consensus of international experts.

### Description of features of a sample of GItools

Guidelines were identified in the Guideline Clearinghouse in June and July of 2012. This resource contains approximately 2,500 guidelines. For feasibility, guidelines were sampled by clinical topic including arthritis, breast cancer, diabetes, stroke, angina, asthma, depression, and prostate cancer. These topics were selected because they are major causes of disability and death worldwide and affect both men and women. Eligible guidelines addressed overall management of these conditions, and were produced within five years by organizations having developed at least 10 guidelines. Full text guidelines were examined, as was the content of corresponding developer web sites to identify information or resources considered to be a GItool. For this analysis, GItools were defined as noted earlier. One research assistant initially identified eligible guidelines in the Guideline Clearinghouse. Two research assistants independently identified potential GItools within guidelines or on corresponding developer web sites. The principal investigator and an RA together reviewed these to confirm they could be considered GItools. Two research assistants independently assessed each GItool for GItool features according to the consensus items generated by the Delphi process. Data were tabulated and summarized to describe the number and type of GItools with features considered desirable.

## Results

### Cross-sectional survey to generate and rate candidate GItool features

The survey was fully completed by 96 respondents from Australia (37.5%), Canada (17.7%), United Kingdom (6.25%), United States (7.3%), The Netherlands (7.3%), various other countries (16.7%), and not indicated (7.3%). Of these, 65.6% were developers, 50.0% implementers, 41.7% researchers, 18.8% other, and 15.6% indicated no position. Of nine items presented, 65% or more of respondents agreed or strongly agreed that seven represented desirable GItool features (Table 2). Of the remaining, one was rated ‘agree’ or ‘strongly agree’ by 63.8% (GItool development is clearly described). Rating was variable for full-scale evaluation of GItools (#8), which did not achieve consensus as a desirable feature (44.7% agree or strongly agree).

**Table 2:** **Cross-sectional survey to generate and rate desirable GItool features (n = 96)**

GItool feature	Rating (n,%)								
	1	2	3	4	5	6	7	Unsure	6 + 7
1. Tool objectives are stated	0	1	0	2	5	22	66	0	88
	0.0	1.0	0.0	2.1	5.2	22.9	65.3	0.0	91.7
2. Target users of tool are identified	0	1	1	4	6	19	65	0	84
	0.0	1.0	1.0	4.2	6.3	19.8	67.8	0.0	87.5
3. Tool development is clearly described	0	1	6	5	20	17	45	2	62
	0.0	1.0	6.3	5.2	20.8	17.7	46.9	2.1	64.6
4. Evidence is cited that underpins tool design, development, content	1	0	4	12	11	19	49	0	68
	1.0	0.0	4.2	12.5	11.5	19.8	51.0	0.0	70.8
5. Quantity and quality of underpinning evidence is described	1	2	2	15	12	22	40	2	62
	1.0	2.1	2.1	15.6	12.5	22.9	41.7	2.1	64.6
6. Development involved pre-testing (gathering stakeholder needs and suggestions by interview, focus group, survey, etc.)	3	2	0	6	13	29	39	4	68
	3.1	2.1	0.0	6.3	13.5	30.2	40.6	4.2	70.8
7. Development involved pilot-testing with stakeholders to assess use and satisfaction, and then improve the tool	1	3	2	3	11	25	46	5	71
	1.0	3.1	2.1	3.1	11.5	26.0	47.9	5.2	74.0
8. Development involved full-scale evaluation with a larger sample of stakeholders to thoroughly/rigorously assess impact	1	2	9	12	24	19	23	6	42
	1.0	2.1	9.4	12.5	25.0	19.8	24.0	6.3	43.8
9. Once implemented, user feedback is prospectively collected to monitor tool use and impact	0	1	3	4	14	30	42	2	72
	0.0	1.0	3.1	4.2	14.6	31.3	43.8	2.1	75.0

No new items were suggested. All features were considered desirable, but several respondents commented on whether it was feasible to develop GItools with all of these features: ‘this is the gold standard, not sure whether it is achievable’ and ‘the criteria are ideal, in practice it will be hard to achieve.’ A few respondents said that developers may not have the resources for pilot-testing or more comprehensive evaluation, and that simple GItools may not require rigorous evaluation. This was corroborated by others who said that (see Table 2) #1 to #5 were essential requirements when developing GItools, #6 and #7 were important but not essential if resources were limited, and #8 would be a lower priority but compensated for by prospective evaluation (#9). A few respondents said that rigorous testing would require considerable time and delay the use of GItools that would otherwise improve care delivery and outcomes, further emphasizing the benefit of making less-rigorously developed GItools available, and the value of prospective evaluation. Views were not uniform. For example, one respondent said that prospective evaluation may not be possible because GItools that were not useful would be rapidly discarded, and another respondent said that implementation of GItools with unknown impact should be actively discouraged.

### Delphi consensus process to confirm desirable GItool features

A total of 31 panelists from 10 countries including Australia, Canada, Germany, New Zealand, Peru, Saudi Arabia, Spain, the United Kingdom, and the United States completed survey #1. Of these, 30 panelists from nine countries complete survey #2. Results are summarized in Table 3. In round #1, 65% of panelists agreed or strongly agreed that 10 of 16 GItool features were desirable, and 8 new features were suggested. In round #2, six features that did not achieve favourable consensus in round #1 were re-rated. Of these, 65% of panelists agreed or strongly agreed with one (prospective collection of user feedback). Of the eight newly suggested features that were rated in round #2, 65% or more of panelists agreed or strongly agreed with one (reporting of context or setting in which tool developed/used). Table 4 lists the final 12 GItool features considered desirable.

**Table 3:** **Delphi survey to confirm desirable GItool featuresGItool feature**

	Round #1	Round #2
1. Tool objectives are stated	28 (90.3)	---
2. Target users of tool are identified	27 (87.1)	---
3. Methods used to develop the tool are clearly described	21 (67.7)	---
4. Instructions are provided on how to use the tool	28 (90.3)	---
5. Conflicts of interest of those involved in tool development are disclosed	19 (61.3)	19 (63.3)
6. Target users informed tool content and format (survey, interview, focus group, committee)	22 (71.0)	---
7. Experts in tool content, and instrument development and design were involved in tool development	19 (61.3)	13 (43.3)
8. A comprehensive literature review was undertaken to inform and assemble tool content	23 (74.2)	---
9. Sources are cited for evidence upon which tool content is based	23 (74.2)	---
10. Quantity and quality of evidence upon which tool content is based is described	20 (64.5)	---
11. The tool was pilot-tested with users and refined based on their feedback prior to implementation	22 (71.0)	---
12. Pilot-testing was rigorous (appropriate sampling, methods)	17 (54.8)	15 (50.0)
13. A description is included of how the tool was evaluated	20 (64.5)	---
14. Tool effectiveness was assessed by full scale evaluation of impact on clinicians and/or patients	15 (48.4)	8 (26.7)
15. Full scale evaluation was rigorous (appropriate sampling, methods)	15 (48.4)	12 (40.0)
16. User feedback about tool use and impact is prospectively collected	15 (48.4)	20 (66.7)
17. The type of tool (domain, subdomain) are specified	---	16 (53.3)
18. The theoretical basis or rationale for the tool is described	---	11 (36.7)
19. Electronic versions are available for computer or mobile device application	---	13 (43.3)
20. Experts in the context/setting in which tool will be used were involved in development	---	18 (60.0)
21. Details of the context/setting in which tool was developed/will be used are described	---	20 (66.7)
22. Success factors/learning based on tool use or evaluation are described	---	14 (46.7)
23. Impact was assessed with rapid cycle testing (*i.e*., PDSA, different from full scale research)	---	7 (23.3)
24. The meaning of full scale evaluation results are interpreted based on an implementation threshold	---	6 (20.0)

**Table 4:** **GItool features considered desirable by the international guideline community**

Component	Definition/Examples
1. Tool objectives are stated	The purpose of the tool is described including intent, use and desired impact or outcome
2. Target users are named	Individuals or groups meant to implement and/or apply the tool are identified
3. Instructions on tool use are provided	Detailed instructions are provided for how to implement and use the tool
4. Methods used to develop the tool are described	Methods used to develop the tool are clearly described, including the process and those involved
5. Tool is based on a comprehensive search of sources for content	A comprehensive literature review was undertaken to inform and assemble tool content and format
6. Evidence upon which tool content is based is described	Quantity and quality of evidence upon which tool content and format is based is described
7. Sources of evidence are cited	Sources are cited for evidence upon which tool content and format are based
8. Context or setting in which tool was developed/will be used are described	Details of the context or setting in which the tool was developed or is meant to be used are described
9. Target users were involved in tool development	Target users were consulted by survey, interview, focus group or as planning committee members
10. Methods used to evaluate the tool are described	Methods used to evaluate the content, format, use and/or impact of the tool are described
11. The tool was pilot-tested with users	The tool was pilot-tested with users and refined based on their input before broad implementation
12. User feedback about tool use and impact is prospectively collected	A mechanism was established to prospectively gather feedback from users about use and impact

Five panelists commented that all features were essential. One panelist said that it was important not to limit access to GItools without all features, noting that many would be adapted and improved by others. Another panelist said that features achieving consensus on desirability could be considered ‘key’ and the rest labeled as ‘nice to have.’ Another panelist said GItools could be made available after pilot testing and that full scale evaluation was better done by those who did not develop the tool. Another respondent said that if GItools were shared in a database, users or developers could prospectively post comments such that the original developers or others could improve the GItool based on this feedback.

### Description of features of a sample of GItools

A search of the Guidelines Clearinghouse identified 149 guidelines on the overall management of arthritis, breast cancer, diabetes, stroke, angina, asthma, depression, and prostate cancer produced within five years by organizations having developed at least 10 guidelines. A search of the content of each guideline and the developer’s web site identified a total of 13 GItools among 149 guidelines (8.7%). This included four Resource planning, seven Implementation, and two Evaluation GItools. Each GItool, the associated guideline and the developer’s web site were searched for information pertaining to each desirable GItool feature (Table 5). Overall, most GItools named target users (92.3%) and described development methods (84.6%); a moderate number of GItools reported or provided objectives (53.8%), instructions (61.5%), search strategy for sources of content (53.8%), sources of content (61.5%), context in which tool was developed or will be used (53.8%), and how target users were involved in development (53.8%); few GItools described underlying evidence (23.1%); methods used to evaluate the GItool (7.7%), or whether prospective feedback about GItool use was gathered (30.8%); and no GItools mentioned pilot-testing of the GItool with target users. Inclusion of this information in GItools across categories of GItools was variable.

**Table 5:** **Features of a sample of GItools**

GItool feature	GItool features			Total n (%)
	Resource planning (4)	Implementation (7)	Evaluation (2)	
1. Tool objectives are stated	2	4	1	7 (53.8)
2. Target users are named	3	7	2	12 (92.3)
3. Instructions on tool use are provided	4	2	2	8 (61.5)
4. Methods used to develop the tool are described	4	5	2	11 (84.6)
5. Tool is based on a comprehensive search of sources for content	4	3	0	7 (53.8)
6. Evidence upon which tool content is based is described	2	0	1	3 (23.1)
7. Sources of evidence are cited	4	2	2	8 (61.5)
8. Context or setting in which tool was developed/will be used are described	4	2	1	7 (53.8)
9. Target users were involved in tool development	4	2	1	7 (53.8)
10. Methods used to evaluate the tool are described	0	0	1	1 (7.7)
11. The tool was pilot-tested with users	0	0	0	0 (0.0)
12. User feedback about tool use and impact is prospectively collected	2	1	1	4 (30.8)

## Discussion

The primary purpose of this research was to characterize the ideal features of GItools. This work is unique because existing instruments and criteria are specific to the development or appraisal of high quality guidelines and not accompanying GItools that are meant to support implementation of guideline recommendations [5]-[8]. The international guideline community including developers, implementers and researchers were engaged in generating a 12-item framework of desirable GItool features. All features were considered important, and one respondent said that implementation or use of GItools with unknown impact should be discouraged. Many respondents said that while ideal, addressing items pertaining to evaluation of GItools may not be feasible for guideline developers given limited time and resources. Pilot-testing with target users was considered important, but full scale evaluation less so. In part, this was due to the feasibility of evaluation, and in part respondents did not want to limit access to GItools. Instead they thought that GItools would likely be adapted and improved upon release, and that a centralized repository should be developed to collect experiences from users that could inform improvement of GItools by developers, researchers or other users. A secondary or related purpose of this research was to describe GItools accompanying a selection of guidelines using the GItool framework. A total of 13 GItools were identified among 149 guidelines on a variety of clinical topics (8.7%). Many did not possess most of the features considered desirable by the international guideline community.

Interpretation and application of these findings may be limited by several factors. We may have failed to identify all possible GItool features, not all individuals with expertise in guideline development and implementation may have been engaged, and we may not have accurately characterized GItools using the framework of desirable features. However, we solicited opinions through G-I-N, which represents and reaches guideline developers, implementers and researchers worldwide, and several individuals independently analyzed GItools for the desirable features. One respondent noted that all items may not be applicable to different types of GItools. Further use of the framework to evaluate GItools of different types is needed to ascertain this, and to either highlight the features relevant to certain types of GItools, or reveal additional features specific to certain types of GItools. The GItool framework reflects the views of guideline developers, implementers and researchers. While some of these individuals may also be guideline users, further validation of the desirable features is needed through direct consultation with target users of guidelines, and by asking guideline users to assess the relevance and use of GItools that do and do not possess the desirable features.

While further validation may be warranted, this research served as a baseline assessment of the current status of GItools and found that the GItools examined lacked many of the features considered desirable by the international guideline community. The resulting framework could therefore function as the basis for evaluating and adapting existing GItools, or developing new GItools that describe not only the objectives and target users, but also provide details about their development, sources of content, evaluation, and potential impact.

Guideline developers may experience a number of barriers in applying the GItool framework. For example, options and instructions for operationalizing each element may be needed. Work is ongoing to translate this framework into an instrument or toolkit by which developers can evaluate and adapt, or develop new GItools. The need for such guidance was confirmed by Schunemann *et al*., who reviewed guideline development and instructional manuals, several of which noted the need to develop or adapt tools or derivative products to provide guidance on how the recommendations can be implemented in practice, but provided limited information on how to achieve that [4]. The GItool framework generated by this research serves as the first step in developing more detailed guidance.

In previous research, guideline developers noted that limited funding challenged guideline implementation [11]-[13]. In this research, though respondents believed that all GItool features were important, they noted that lack of funding would limit their capacity to address GItool framework elements related to pilot-testing or evaluating GItools, or prospectively gathering user feedback. It appears that some evaluation of GItools in the form of pilot-testing with target users prior to release is considered warranted. Pilot-testing can be variable in scope and rigour. For example, pilot-testing could consist of review by a few target users, or use of the GItool for a period of time by a few users, or more rigorous evaluation among a large number of different types of target users. Further analysis of the extent and methods of pilot-testing needed for different types of GItools may be needed. This information could help guideline developers plan, prioritize, and budget for pilot-testing.

An alternative means for addressing the barrier of limited funding is to draw on the principles and practices of action research or integrated knowledge translation, which could be used to establish researcher-developer partnerships for the purpose of evaluating GItools through pilot-testing [29],[30]. Participants thought that shared development of GItools was a feasible way to more rigorously evaluate and improve GItools. This could be achieved by releasing GItools and then prospectively collecting feedback from users upon which developers or others could improve them. While we developed a repository of GItools (http://giranet.org), this was done as a means of providing examples of different types of GItools. Growing and sustaining such a database may not be possible. It may be more feasible to integrate the GItool framework or a mechanism for feedback about GItools into existing repositories of guidelines.

We identified few GItools (8.7%) among the guidelines that were examined despite sampling guidelines on a range of topics. The number of guidelines examined represented approximately 6.0% of the guidelines in the National Guideline Clearinghouse so additional guidelines could be sampled to identify a larger number of GItools that could be evaluated according to the GItool framework. However, it appears that few GItools possess many of the features considered desirable. Broad dissemination of the GItool framework among developers may be needed to prompt the adaptation or development of GItools. In this regard, several questions remain to be addressed by further research. For example, should all guidelines be accompanied by one or more GItools? Which types of GItools are most suitable for different guidelines? Ongoing research could also explore the challenges faced by developers when adapting or developing GItools so that suitable solutions can be identified. Ultimately, the impact of GItools featuring desirable characteristics could be evaluated.

While many questions requiring additional research are posed here, the GItool framework generated through consultation with the international guideline community represents the current gold standard for assessing or developing GItools. Practical application of the GItool framework may lead to the inclusion of higher quality GItools with more guidelines that may support broader and more consistent implementation and use of guidelines by target users.

## Conclusions

Consultation with and engagement of international guideline developers, implementers and researchers generated a 12-item framework of desirable features of GItools. Among a sample of guidelines, few GItools were identified. Examination of GItools with this framework found that few possessed features considered desirable by the guideline community. Further research is needed to validate the framework, develop and implement instruments by which developers can apply the framework, and specify which guidelines should be accompanied by GItools.
